# The Role of High-Density Lipoproteins in Reducing the Risk of Vascular Diseases, Neurogenerative Disorders, and Cancer

**DOI:** 10.1155/2011/496925

**Published:** 2010-12-23

**Authors:** Donovan McGrowder, Cliff Riley, Errol Y. St. A. Morrison, Lorenzo Gordon

**Affiliations:** ^1^Department of Pathology, Faculty of Medical Sciences, University of the West Indies, Mona Campus, Kingston 7, Jamaica; ^2^College of Health Sciences, University of Technology, 237 Old Hope Road, Kingston 6, Jamaica; ^3^Principal's Office, University of Technology, 237 Old Hope Road, Kingston 6, Jamaica; ^4^Department of Medicine, Faculty of Medical Sciences, University of the West Indies, Kingston 7, Jamaica

## Abstract

High-density lipoprotein (HDL) is one of the major carriers of cholesterol in the blood. It attracts particular attention because, in contrast with other lipoproteins, as many physiological functions of HDL influence the cardiovascular system in favourable ways unless HDL is modified pathologically. The functions of HDL that have recently attracted attention include anti-inflammatory and anti-oxidant activities. High anti-oxidant and anti-inflammatory activities of HDL are associated with protection from cardiovascular disease. Atheroprotective activities, as well as a functional deficiency of HDL, ultimately depend on the protein and lipid composition of HDL. Further, numerous epidemiological studies have shown a protective association between HDL-cholesterol and cognitive impairment. Oxidative stress, including lipid peroxidation, has been shown to be the mediator of the pathologic effects of numerous risk factors of Alzheimer's disease. Lifestyle interventions proven to increase HDL- cholesterol levels including “healthy” diet, regular exercise, weight control, and smoking cessation have also been shown to provide neuro-protective effects. This review will focus on current knowledge of the beneficial effects of HDL-cholesterol as it relates to cardiovascular diseases, breast and lung cancers, non-Hodgkin's lymphoma, as well as its neuroprotective potential in reducing the risk of Alzheimer's disease and dementia.

## 1. Introduction

### 1.1. Composition of HDL

Plasma high-density lipoproteins (HDLs) are small, dense, and spherical lipid-protein complexes and are normally considered to consist of those plasma lipoprotein particles which fall into the density range of 1.063–1.210 g/mL. HDL particles are composed of an outer layer containing free cholesterol, phospholipid, and various apolipoproteins (Apo), which covers a hydrophobic core consisting primarily of triglycerides and cholesterol esters [1]. The major proteins are Apo A-I (Mr 28,000) and Apo A-II (Mr 17,000). Apo A-I, the primary protein constituent of these particles, accounts for about 60% of the protein content of HDL. Apo A-I is synthesized in the intestines and liver and is thought to be largely responsible for the antiatherogenic effects of HDL. Some HDL particles carry only Apo A-I, whereas others contain both Apo A-I and Apo A-II [2]. 

Other apolipoprotein species found in HDL particles include Apo A-IV, Apo C (C-I, C-II, and C-III), and Apo E. Several subtypes of HDL particles have been identified on the basis of density, electrophoretic mobility, particle size, and apolipoprotein composition [3]. Differences in particle size are mainly the result of the number of apolipoprotein particles and the volume of the cholesterol ester in the core of the particle [3]. 

HDL can be classified into larger, less dense HDL_2_ or smaller, denser HDL_3_ which falls within the density ranges 1.063–1.125 and 1.125–1.210 g/mL, respectively. Although the major proportion of HDL is normally present in HDL_3_, individual variability in HDL levels in human populations usually reflects different amounts of HDL_2_ [4]. HDL_2_ is richer in particles containing Apo A-I without Apo A-II, whereas HDL_3_ is richer in particles containing both Apo A-I and Apo A-II [5].

HDL particles have multiple biological effects that could contribute to their antiatherothrombotic action including anti-inflammatory, antioxidant, and profibrinolytic activities [2]. The health benefits of HDL as documented in the literature are many. The purposes of this paper are to review current knowledge on the beneficial effects of HDL-cholesterol as it relates to (i) cardiovascular diseases, (ii) breast and lung cancers as well as non-Hodgkin's lymphoma, and (iii) Alzehemier's disease and dementia.

## 2. HDL-Cholesterol and Coronary Heart Disease

### 2.1. Relationship between HDL-Cholesterol and Coronary Heart Disease

Numerous prospective cohort studies support a powerful inverse correlation between circulating HDL-cholesterol and coronary risk among patients with normal or elevated low-density lipoprotein- (LDL-) cholesterol [6, 7]. Low HDL-cholesterol levels increase the risk of coronary heart disease (CHD) and analysis of the largest US epidemiologic studies including the Framingham Heart Study, the Lipid Research Clinics (LRC) Primary Prevention Trial, the LRC Prevalence Mortality Followup Study, and the Multiple Risk Factor Intervention Trial (MRFIT) substantiated that, on average, each one (1) mg/dL increase in HDL-cholesterol reduces CHD risk by 2% in men and 3% decrease in women [8]. The Framingham Heart Study have supported the role of low HDL-cholesterol as an independent risk factor for coronary artery disease (CAD) and demonstrated that subjects with the highest HDL-cholesterol levels exhibit the lowest risk of developing CAD. Further, based on 14 years of surveillance, the Framingham Study showed that there is an inverse relation between HDL-cholesterol and the incidence of CAD [9]. Subsequent follow-up revealed that study participants at the 80th percentile of HDL-cholesterol were found to have half the risk for developing CAD when compared with subjects at the 20th percentile of HDL-cholesterol [9]. Among patients aged 50 to 80 years without ischaemic heart disease (IHD), after a mean follow-up of 4 years, the risk of myocardial infarction (MI) or IHD death decreased by 25% for every 10 mg/dL increase in HDL-cholesterol [9]. 

The Israeli Ischemic Heart Disease Study examined the CHD and all-cause mortality of some 8000 Israeli men aged 42 years and older and found that subjects with low total cholesterol and high HDL-cholesterol had the lowest rates of CAD-associated morbidity and mortality [10]. The Physicians' Health Study showed that subjects with low HDL-cholesterol had increased risk for CAD. Both HDL-cholesterol and HDL_2_ levels were associated with a substantially decreased risk of MI, but the HDL_3_ level was the strongest predictor, the relative risk being 0.3 [11]. Levels of Apo A-I and Apo A-II were also associated with decreased risk [11]. The Prospective Cardiovascular Munster Study (PROCAM) demonstrated that subjects with HDL-cholesterol levels ≤35 mg/dL were at 3 times greater risk for CAD compared with those who had HDL-cholesterol levels ≥35 mg/dL [12].

### 2.2. HDL-Cholesterol as a Therapeutic Target

Reductions in LDL-cholesterol using statins form the cornerstone of treatment of hyperlipidemia to reduce cardiovascular morbidity and mortality. Low serum concentrations of HDL-cholesterol are one of the major risk factors for adverse events related to coronary atherosclerosis [8] and are highly prevalent among patients with acute coronary syndromes [13]. Niacin is the most effective therapy available for the treatment of low HDL-cholesterol, with a nonlinear dose-related increase in HDL-cholesterol of approximately 20% observed with modest drug doses (approximately 1 g per day) [14, 15]. In the Collaborative Atorvastatin Study Group which involved a randomized trial of the effects of atorvastatin and niacin in patients with combined hyperlipidemia or isolated hypertriglyceridemia, atorvastatin increased HDL-cholesterol by 4% and niacin by 25% [16]. The specific mechanism of action of niacin is not well understood, but it appears to reduce Apo B secretion, thereby lowering both very low density lipoprotein (VLDL) and LDL, increasing Apo A-I, and lowering lipoprotein(a) [17]. The ARBITER (Arterial Biology for the Investigation of the Treatment Effects of Reducing Cholesterol-) 2 study examined the effects of niacin added to background statin therapy on HDL-cholesterol and carotid intima-media thickness (CIMT), a validated surrogate cardiovascular end point [18]. After 12 months of therapy, extended-release niacin increased HDL-cholesterol by 21% and decreased the rate of CIMT progression [18]. Therefore, the addition of extended-release niacin to statin therapy slowed the progression of atherosclerosis among individuals with known coronary heart disease and moderately low HDL-cholesterol [18]. 

In the Helsinki Heart Study and the Veterans Affairs High-Density Lipoprotein Cholesterol Intervention Trial (VA-HIT), treatment with gemfibrozil was associated with reduction in CVD events [19, 20]. The Helsinki Heart Study examined the effect of gemfibrozil on lipid parameters and cardiovascular outcomes among high-risk middle-aged men without IHD and with primary dyslipidemia [19]. After 5 years of follow-up, treatment with gemfibrozil increased HDL-cholesterol by 11%, decreased LDL-cholesterol and triglycerides by 11% and 35%, respectively, and reduced the incidence of MI and IHD death by 34%. The study demonstrated that for every one (1) mg/dL increase in HDL-cholesterol, the CHD risk decreased by 2% to 3%, independent of changes in LDL-cholesterol [19]. In the VA-HIT cohort, gemfibrozil was associated with a significant 22% reduction in the risk of fatal and nonfatal CHD, and it significantly reduced the relative risk of investigator-designated stroke [20]. Some evidence indicates that the benefits seen in the VA-HIT cohort may be partly due to additional effects of fibrates on apolipoprotein synthesis and lipoprotein metabolism [21, 22]. In contrast, the results of studies using other fibrates such as bezafibrate and fenofibrate have been negative. Bezafibrate has no effect on the incidence of CHD and of stroke combined but may reduce the incidence of nonfatal coronary events, particularly in those aged <65 years at entry, in whom all coronary events may also be reduced [23]. Fenofibrate did not significantly reduce the risk of the primary outcome of coronary events; however, it did reduce total cardiovascular events, mainly due to fewer non-fatal MIs and revascularisations [24].

### 2.3. Mechanism Involved in the Beneficial Effects of HDL-Cholesterol

The beneficial role of HDL-cholesterol in reducing atherosclerotic disease burden is complex and likely involves multiple mechanisms and biochemical pathways. The primary mechanism by which HDL-cholesterol is thought to reduce cardiovascular risk is reverse cholesterol transport, a pathway for removing free cholesterol from the macrophage in the arterial wall and returning it to the liver for excretion into bile [7, 25]. HDL-cholesterol-mediated reductions in cardiovascular risk could occur through a variety of other pathways, including raising HDL-cholesterol levels (by decreasing catabolism or increasing synthesis) and having a larger number of particles available with which to antagonize proatherogenic stimuli [26]. Collectively, HDL and its components [methionine resides in Apo A-I, paraoxonase 1 (PON1), platelet activating factor-(PAF-) acetylhydrolase, and other antioxidant enzymes] can reduce oxidized lipid species in LDL particles, rendering them less atherogenic. This may help prevent atherosclerosis, acute coronary syndromes, and restenosis after coronary angioplasty [27]. 

HDLs have antioxidant activity through the antioxidative properties of Apo A-I, and the presence of enzymes such as PON1, glutathione-peroxidase, and PAF-acetylhydrolase. Paraoxonases 1 is an HDL-associated enzyme with antioxidant properties. There are data suggesting that the direct antioxidant effect of HDL on LDL oxidation, measured as a reduction in lipid peroxides, is likely mediated by PON1 [28]. Impaired PON1 activity was found to be closely linked to increased risk of cardiovascular mortality [29]. Moreover, PON1 activity is decreased in patients with type 2 diabetes with [30] or without CAD [31].

HDLs seem to exert a beneficial effect on endothelium function by modulating the activity and/or production of a number of molecules [such as nitric oxide (NO), prostacyclin (PGI_2_), endothelin, and PAF], and affecting vascular tone and thrombogenicity [32]. Plasma HDL-cholesterol levels have been found to be an independent predictor of NO-dependent peripheral vasodilation in healthy individuals [33], hyperlipidaemic and diabetic patients [34, 35], and patients with CHD [36]. Further, HDLs display an antithrombotic effect by inhibiting platelets aggregation, reducing von Willebrand factor levels, and enhancing the activity of activated protein C and S [37]. 

## 3. HDL-Cholesterol and Risk of Cancer

### 3.1. HDL-Cholesterol and Breast Cancer Risk

Boyd and McGuire in 1990 proposed that women with high levels of HDL-cholesterol may have an increased risk of breast cancer [38]. This hypothesis by these authors based on the observation that many known and suspected breast cancer risk factors are associated with increased levels of HDL-cholesterol, and experimental studies that suggest that HDL-cholesterol has a role in the proliferation of breast tumor cells *in vitro *[39, 40]. Further, HDL-cholesterol could play a role in carcinogenesis through its influence on cell cycle entry, via a mitogen-activated protein kinase-dependent pathway [41] or regulation of apoptosis [42]. Therefore, *in vitro *studies that show that HDL-cholesterol promotes the growth of breast cancer cell lines in tissue culture suggest that a direct, causal effect is biologically plausible [40, 43]. However, it is unknown if a similar effect on tumor cells occurs *in vivo.*

Two studies have addressed prospectively the association of HDL-cholesterol and breast cancer incidence [44, 45]. Hoyer and Engholm investigated the association between serum lipids and breast cancer risk in a cohort of 5,207 Danish women, who participated in The Glostrup Population Studies between 1964 and 1986 [44]. They found an inverse association between serum HDL-cholesterol and risk of breast cancer, with HDL-cholesterol being significantly lower among women who developed breast cancer during follow-up [44]. Gaard and colleagues in a prospective study examined the relationship between blood lipids and breast cancer risk in pre and postmenopausal Norwegian women. They found no association between breast cancer risk and blood lipids in the total population during 7 to 13 years of follow-up [45].

Several studies have reported lower levels of HDL-cholesterol in breast cancer patients than in control subjects [46–49]. HDL-cholesterol levels were found to be significantly lower in pre- and postmenopausal women with benign and malignant breast disease [46]. Kokoglu et al. reported significant increase in triglycerides and VLDL-cholesterol and decrease in HDL- and LDL-cholesterol levels in patients with stage IV disease when compared to those with stage I breast cancer [47]. A decrease in HDL-cholesterol, especially in the HDL_2_ subfraction, was observed in newly diagnosed breast cancer patients. Besides, HDL particle from these patients showed increased Apo A-I/HDL-cholesterol ratio [47]. Further, there are several reports that have shown that tumor progression from localized to metastatic disease is associated with declining HDL-cholesterol levels [43, 47].

Two population-based screening surveys involving Norwegian women found that low HDL-cholesterol, as part of the metabolic syndrome, is associated with increased post-menopausal breast cancer risk, and HDL-cholesterol association was confined to women in the heavier subgroup with body mass index (BMI) ≥25 kg/m^2^ [50]. The risk of post-menopausal breast cancer among overweight and obese women in the highest serum HDL-cholesterol quartile was one-third the risk of women in the lowest serum HDL-cholesterol quartile. According to the authors, these findings suggest an interaction between metabolic disturbances (i.e., overweight or obesity and low-serum HDL-cholesterol) in postmenopausal breast carcinogenesis [50]. In a nested case-control study by Moorman et al., there was a positive association between HDL-cholesterol in serum stored for more than 20 years and postmenopausal breast cancer risk. Further, an inverse association with premenopausal breast cancer risk was observed [51].

### 3.2. HDL-Cholesterol and Lung Cancer Risk

Cancer patients often present altered serum lipid profile including changes of HDL-cholesterol level. Case-control studies of newly diagnosed lung cancer have shown that HDL-cholesterol levels are reduced in lung cancer cases relative to levels observed in the control groups [52–55]. Siemianowicz et al. evaluated serum level of HDL-cholesterol in patients with squamous cell and small cell lung cancer and its dependence on histological type and clinical stage of lung cancer. They found that there were no statistically significant differences of HDL-cholesterol level between the histological types or between clinical stages of each histological type of lung cancer [52]. Decreased serum HDL-cholesterol was also reported in advanced nonresectable lung cancer patients [53]. Kucharska-Newton et al. examined prospectively the association of baseline plasma HDL-cholesterol levels with incidence of lung cancer in subject of the Atherosclerosis Risk in Communities (ARIC) cohort. They found relatively weak inverse association of HDL-cholesterol with lung cancer that was dependent on smoking status, even after exclusion of cases diagnosed within 5 years of baseline [56]. In the Alpha-Tocopherol, Beta-Carotene Cancer Prevention (ATBC) Study cohort, higher HDL-cholesterol levels were related to modestly decreased risk of cancer overall; this association remained after excluding cases diagnosed during the first 15 years of follow-up [57].

Biological mechanisms that might link low plasma levels of HDL-cholesterol with cancer are not well understood [38, 55]. The function of HDL-cholesterol in reverse cholesterol transport is important in development of atherosclerosis; however, it is not obvious how this function of HDL-cholesterol could influence carcinogenesis [58]. Further, HDL regulation of cell cycle entry through a mitogen activated protein kinase-dependent pathway [41] and apoptosis [42], modulation of cytokine production, and antioxidative function [59] has been considered and is biologically plausible. The association between higher HDL-cholesterol and lower overall cancer incidence observed in the ATBC cohort is biologically plausible, as HDL- cholesterol has anti-inflammatory properties [59]. However, it is also plausible that this association reflects the effect of factors that are associated with both HDL-cholesterol and risk of cancer, such as inflammation. Inflammation reduces HDL-cholesterol [60] and likely increases risk of lung cancer [61].

### 3.3. HDL and Lymphoma Risk

Lipoprotein abnormalities seen in patients with inflammatory diseases are thought to develop secondary to circulating cytokines and the accompanying acute-phase response. Lymphoma patients often exhibit abnormal lipid metabolism. Clinical studies of lymphoma patients have reported lipid abnormalities that are similar to the dyslipidemia observed in inflammatory and infectious diseases [62, 63]. Further, a decrease in circulating HDL-cholesterol may occur during lymphomagenesis, reflecting underlying etiology such as inflammation.

Spiegel et al. investigated plasma lipids and lipoproteins at presentations in 25 patients with acute leukemia and non-Hodgkin's lymphoma (NHL). They found that all patients demonstrated an abnormality in at least one plasma lipid fraction and most exhibited a predictable pattern of lipid alterations that consisted of extremely low levels of HDL-cholesterol, elevated triglyceride, and elevated VLDL [62]. The degree of lipid abnormality was directly related to the underlying tumor burden and particularly to the presence of bone marrow involvement [62].

 To evaluate the etiologic involvement of HDL-cholesterol in lymphomagenesis, Lim et al. investigated the association between prediagnostic serum HDL-cholesterol levels and the subsequent development of NHL during 17 years of follow-up of Finnish male smokers in the Alpha-Tocopherol Beta-Carotene (ATBC) Cancer Prevention Study cohort [64]. They observed an inverse association between HDL-cholesterol and NHL, which changed with length of follow-up. High HDL-cholesterol was associated with lower risk of all NHLs during the first 10 years, but not with diagnoses during later follow-up [64]. Therefore, low circulating levels of HDL-cholesterol in lymphoma patients may occur before the clinical onset of cancer and may serve as a marker for inflammation-induced lymphomagenesis, rather than a consequence of lymphoma-induced acute-phase responses [63].

Studies have shown that chronic inflammation is known to reduce both serum HDL-cholesterol levels and its anti-inflammatory properties [65, 66]. Low HDL-cholesterol, therefore, may be a marker for the severity of systemic inflammation and inflammation-induced NHL risk. Conversely, high HDL-cholesterol itself may be protective against NHL. High-density lipoprotein-cholesterol seems to modulate inflammatory responses independent of non-HDL cholesterol levels [32] by suppressing chemotactic activity of monocytes and lymphocytes [67, 68] and inhibiting cytokine-induced expression of endothelial cell adhesion molecules. Further, HDL-cholesterol may protect the integrity of lymphocytes from oxidative damage [25, 69]. 

## 4. HDL Protects the Aging Brain

### 4.1. HDL, Alzheimer's Disease and Dementia

Over the past decade, abnormalities in brain lipid metabolism are increasingly recognized to be intimately related to the pathogenesis of such major neurodegenerative disorders as Alzheimer's disease (AD) and vascular dementia (VD). With respective frequencies of 70% and 15% of all dementias, AD and VD are the most common forms of dementia which are typically preceded by less dramatic cognitive decline, including decline in memory [70]. Both AD and atherosclerosis develop in parallel with the aging process and as such share a number of risk factors, including the presence of type 2 diabetes and elevated levels of total cholesterol and Lp(a) in midlife, thereby suggesting common pathophysiological pathways [71]. 

Cerebrovascular and Alzheimer's pathological changes frequently coincide in cases of dementia and may act synergistically in producing cognitive decline [72]. Lipoproteins such as HDL may influence neurodegeneration as a carrier of cholesterol. The extracellular deposition of amyloid beta (A*β*) in senile plaques constitutes one of the defining hallmarks of AD. There is evidence that brain cholesterol is related to amyloid metabolism in the brain and that disruption of cholesterol homeostasis in AD is linked to A*β* pathology [73]. Amyloid beta binds to HDL, maintaining its solubility in cerebrospinal fluid (CSF) and plasma. This HDL-A*β* interaction prevents the deposition of A*β* into the brain and can serve as a marker for neurodegenerative disease [74].

Oxidative stress, including lipid peroxidation, has been shown to be the mediator of the pathologic effects of numerous risk factors of AD [75]. Lifestyle interventions proven to increase HDL-cholesterol levels [76] including “healthy” diet, regular exercise, weight control, and smoking cessation have also been shown to provide neuroprotective effects [77, 78]. Moreover, elevated HDL-cholesterol levels potentially mediated by low cholesterol ester transfer protein (CETP) activity are also associated with longevity, improved cognition, and dementia-free survival [79]. Since HDL is the lipoprotein responsible for the efflux of cholesterol within cells of the brain, it may be that deficient levels or dysfunction of HDL-cholesterol may contribute to certain tauopathies or dysgenesis of synaptic processes, such that individuals with dyslipidemia may be more susceptible to neurodegenerative disease.

Singh-Manoux et al. in The Whitehall II Study examined the relationship between fasting serum lipids and short-term verbal memory in middle-aged adults. They found that low HDL-cholesterol and decreasing levels over a 5-year follow-up period were associated with poor memory and decline in memory, respectively, [80]. Further, according to these authors, there are complex and variable biochemical mechanisms potentially linking HDL to AD. The different neuroprotective properties of HDL include accelerated maturation of synapses, maintenance of synaptic plasticity, improved metabolism of A*β*, increase in hippocampal volume, and anti-inflammatory and antioxidative activities [80]. Further, brain HDL can suppress A*β* production by decreasing cellular cholesterol through the activation of reverse cholesterol transport mediated by ABC transporters [81]. HDL can directly bind excess A*β* and thereby inhibit its oligomerisation [82]. Oligomerisation represents a major step in the transformation of the monomeric nontoxic peptide to the aggregated neurotoxic form that can account for memory impairment [83]. HDL may also remove A*β* that accumulates in the vessel wall during the course of VD; by analogy with reverse cholesterol transport, such a process can be termed “reverse amyloid transport” [84]. In decreasing oxidative stress, HDL directly decreased A*β* production, as oxidative stress induces enhanced production of A*β* as a potentially protective response [85]. Further, HDL can act on astrocytes to attenuate a local inflammatory reaction. 

High-density lipoprotein particles traffic cholesterol in the brain and are related to cholesterol metabolism, which may play an important role in A*β* metabolism and deposition in the brain [86]. HDL-cholesterol is critical for the maturation of synapses and the maintenance of synaptic plasticity [87]. High-density lipoprotein-cholesterol has been found to be associated with hippocampal volume and dementia [88]. 

Next to AD, VD is the second most common form of dementia in the elderly, yet few specific risk factors have been identified. Dementia occurs late in life, but it is increasingly recognized that there is a long preclinical phase characterized by progressive neuropathological changes that become clinically detectable later. The “life-long” view of dementia stresses the importance of vascular risk factors in midlife [89]. Different studies have investigated the relationship between lipid levels and the risk of VD. Many of them found an association with decreased levels of HDL-cholesterol. Zuliani et al. found in a cross-sectional study that low HDL-cholesterol levels are associated with VD, but not with late-onset AD in a sample of 60 older subjects with VD compared with 54 controls [90]. Kuriyama et al. reported lower HDL-cholesterol levels in 44 patients with sporadic late-onset AD and 43 patients with VD compared with controls [91]. The Apo A-II level was disproportionally decreased in the patient groups. Further, the authors found that serum Apo A-II may involve the pathological process in the patients with senile dementia [91]. Muckle and Roy found significantly lower HDL-cholesterol levels in 5 men with multi-infarct dementia than in 12 men with senile dementia of the Alzheimer type [92]. 

In a population-based study involving 561 subjects 85 years old, Mini-Mental State Examination (MMSE) scores were significantly lower in subjects with low HDL-cholesterol. Further, in a comparison of subjects with low HDL-cholesterol with subjects with high HDL- cholesterol, the odds ratio for dementia was 2.3. Thus, low HDL-cholesterol is associated with cognitive impairment and dementia [93]. In the Northern Manhattan Stroke Study, Sacco et al. found that high HDL-cholesterol levels were related to a lower risk of stroke [94]. Increased HDL-cholesterol levels were associated with reduced risk of ischemic stroke in the elderly and among different racial or ethnic groups. This supports HDL-cholesterol as an important modifiable stroke risk factor [94].

However, most longitudinal studies have failed to detect similar associations; HDL-cholesterol levels have not been associated with AD [95–97], vascular dementia [96, 97], or dementia [97]. Romas et al. found no association between HDL and either prevalent or incident AD [95]. In a prospective community-based cohort study of white, African American, and Caribbean Hispanic elderly, Reitz et al. found a weak relation between HDL-cholesterol levels and the risk of VD [96]. Further, in a population-based cohort study, there was no association between HDL-cholesterol in late life and subsequent risk of AD or dementia [97]. A possible explanation for the contradictory findings in the literature is that lipid levels tend to change as people become older [98]. This pattern can be partly explained by physiologic aging, unintentional or voluntary changes in lifestyle (due to increased public health awareness and initiation of interventional programs meant to lower cholesterol levels), selective mortality, or clinical-subclinical disease [99].

Another possible explanation for the conflicting epidemiologic associations between HDL-cholesterol and dementia is that other constituents of HDL-cholesterol such as Apo A-I may be more biologically relevant in AD or VD pathology and may therefore represent a more accurate dementia risk factor. In a case-control study of 45 AD patients and 79 controls, levels of Apo A-I were lower in Japanese patients with late-onset nonfamilial AD [100]. The reductions in Apo A-I levels were observed in both carriers and noncarriers of the e4 allele [100]. Also, in a case-control study of 98 AD patients and 59 elderly controls, serum Apo A-I levels were significantly lower in AD patients [101]. An Apo A-I cutoff value of 1.50 g/L could distinguish between the two groups with a sensitivity of 71 percent and a specificity of 69 percent. Apo A-I levels were highly correlated with MMSE scores and were significantly and consistently lower in AD patients, independent of e4 status [101]. In a population sample of Japanese-American men, lower Apo A-I levels were associated with increased dementia risk more than a decade later. The associations seemed similar for AD and VD and were present over and above adjustments for HDL cholesterol and triglyceride levels [102].

## 5. Conclusion

It is clearly established that low HDL-cholesterol levels are a major independent risk factor for CVD. Further, large cohort studies have identified HDL-cholesterol as a strong, independent, and inverse predictor of risk of CHD. The recent advances in understanding the mechanisms by which HDL acts to reduce cholesterol content in vessel walls have suggested a number of therapeutic strategies for reducing CHD risk by augmenting this activity. Currently available drugs that act to increase HDL-cholesterol concentrations include the statins, fibrates, and niacin. However, although studies such as the Collaborative Atorvastatin Study Group, the ARBITER-2 study, the Helsinki Heart Study, and the VA-HIT provide circumstantial evidence that therapeutic intervention results in the elevation of HDL-cholesterol which translates into beneficial outcomes such as reduced CVD risk, this is not definitive. 

Studies have suggested an association between high HDL-cholesterol levels and the epidemiology of breast and lung cancer risk. High HDL-cholesterol is associated with low risk of developing NHL in a dose-responsive manner. Further studies on the potential role of HDL-cholesterol as an early indicator or even as an etiologic factor of these cancers will be of substantial importance to public health considering the high prevalence of low HDL-cholesterol in developed and developing countries. 

Low HDL-cholesterol is common in adults and contributes to high rates of cardiovascular disease and may be linked to subsequent neurodegenerative and neurovascular diseases. Numerous epidemiological studies have shown a protective association between HDL-cholesterol and cognitive impairment and dementia. There are a number of studies that have shown an association between low levels of HDL-cholesterol and the risk of AD and VD. Therefore, the modulation of HDL function and its concentrations in serum will significantly impact future approaches to the management of cardiovascular diseases, cancers, and neurodegenerative and neurovascular diseases such as AD and VD in both the primary and secondary prevention settings.
